# A Conditional Mutation in *SCD1* Reveals Linkage Between PIN Protein Trafficking, Auxin Transport, Gravitropism, and Lateral Root Initiation

**DOI:** 10.3389/fpls.2020.00910

**Published:** 2020-07-07

**Authors:** Carole L. Gibson, Jonathan W. Isley, Tanya G. Falbel, Cassie T. Mattox, Daniel R. Lewis, Kasee E. Metcalf, Gloria K. Muday

**Affiliations:** ^1^Department of Biology, Center for Molecular Signaling, Wake Forest University, Winston-Salem, NC, United States; ^2^Department of Bacteriology, University of Wisconsin, Madison, WI, United States

**Keywords:** SCD1, gravitropism, lateral root, auxin transport, PIN2, *Arabidopsis thaliana*

## Abstract

Auxin is transported in plants with distinct polarity, defined by transport proteins of the PIN-formed (PIN) family. Components of the complex trafficking machinery responsible for polar PIN protein localization have been identified by genetic approaches, but severe developmental phenotypes of trafficking mutants complicate dissection of this pathway. We utilized a temperature sensitive allele of *Arabidopsis thaliana SCD1* (*stomatal cytokinesis defective1*) that encodes a RAB-guanine nucleotide exchange factor. Auxin transport, lateral root initiation, asymmetric auxin-induced gene expression after gravitropic reorientation, and differential gravitropic growth were reduced in the roots of the *scd1-1* mutant relative to wild type at the restrictive temperature of 25°C, but not at the permissive temperature of 18°C. In *scd1-1* at 25°C, PIN1- and PIN2-GFP accumulated in endomembrane bodies. Transition of seedlings from 18 to 25°C for as little as 20 min resulted in the accumulation of PIN2-GFP in endomembranes, while gravitropism and root developmental defects were not detected until hours after transition to the non-permissive temperature. The endomembrane compartments that accumulated PIN2-GFP in *scd1-1* exhibited FM4-64 signal colocalized with ARA7 and ARA6 fluorescent marker proteins, consistent with PIN2 accumulation in the late or multivesicular endosome. These experiments illustrate the power of using a temperature sensitive mutation in the gene encoding SCD1 to study the trafficking of PIN2 between the endosome and the plasma membrane. Using the conditional feature of this mutation, we show that altered trafficking of PIN2 precedes altered auxin transport and defects in gravitropism and lateral root development in this mutant upon transition to the restrictive temperature.

## Introduction

The process of auxin transport drives many aspects of plant development, including primary root elongation, lateral root development, and asymmetric growth in response to gravity and light gradients (Muday and DeLong, 2001; Vieten et al., 2007; Zazimalova et al., 2010). Auxin (indole-3-acetic acid; IAA) is transported long distances in two polarities in two root cell layers (Muday and DeLong, 2001). Cell to cell IAA transport is mediated by AUX/LAX influx carriers and by efflux carriers of the PIN and ABCB families (Zazimalova et al., 2010). Directionality is controlled by asymmetric localization of PIN proteins at the plasma membrane (Wisniewska et al., 2006; Adamowski and Friml, 2015). In *Arabidopsis* roots, PIN1 is localized to the stele and implicated in auxin transport toward the root apex (Galweiler et al., 1998), which is now called rootward transport and this auxin flow is required for lateral root development (Reed et al., 1998; Lavenus et al., 2013). In contrast, PIN2 is localized to the epidermal and cortical cell layers of roots and mediates shootward transport from the root apex (Muller et al., 1998), which is integral to root elongation and gravitropism (Rashotte et al., 2000). PIN3 is localized to the columella cells at the root tip, where the angle of the gravity vector is perceived, and in the vascular cylinder, consistent with functions in gravitropism and root development, respectively (Friml et al., 2002; Harrison and Masson, 2007).

The polar distribution of the PIN proteins is dynamic (Barbosa and Schwechheimer, 2014; Weller et al., 2017). Targeting of PIN proteins to the membrane in response to stimuli may be an important mechanism to allow changes in auxin transport and dependent growth processes (Paciorek et al., 2005; Barbosa and Schwechheimer, 2014). PIN proteins travel through the endosome to the plasma membrane after *de novo* synthesis through a highly regulated process (Jasik et al., 2013, 2016) involving the RAB and ARF families of small G proteins. GNOM, a well-studied ARF-GEF and target of the drug Brefeldin A, is critical in targeting of PIN1 and PIN2 between the endosome and the plasma membrane (Geldner et al., 2001, 2004, 2003). Although genetic approaches have identified pathways for targeting PIN proteins (Adamowski and Friml, 2015), non-conditional mutants with impaired auxin transport, such as *gnom*, have profound developmental phenotypes (Geldner et al., 2003). This complicates determination of whether altered auxin transport causes developmental phenotypes or the converse.

This study used a conditional *scd1-1* mutant to test whether auxin transport trafficking defects precede growth or developmental phenotypes. The SCD1 protein has a DENN domain, found exclusively in RAB-GEFs (Yoshimura et al., 2010; Marat et al., 2011). SCD1 and an associated protein, SCD2, have been shown to complex with a RabE1 at the exocyst in *Arabidopsis* roots (Mayers et al., 2017), where it plays a role in exocytosis and recycling of membrane proteins. Three *scd1* mutant alleles have been described: *scd1-1*, *scd1-2*, and *scd1-3* (Falbel et al., 2003). *scd1-2* and *scd1-3* are loss of function mutants with altered guard cell development, and profound root growth and developmental phenotypes. However, the temperature sensitive *scd1-1* allele, displays these phenotypes only at the restrictive temperature of 25°C (Falbel et al., 2003).

This study utilizes the unique features of a temperature sensitive *scd1-1* mutant allele to demonstrate that the growth and developmental effects in *Arabidopsis* roots at the restrictive temperature are tied to altered auxin transport protein targeting. The developmental and auxin transport defects can be induced by transition to the restrictive temperature and reversed by return to the permissive temperature. We report the effects of these treatments on polar auxin transport and dependent physiological processes including lateral root development, gravity response, and root elongation. At the restrictive temperature, PIN2 fused to GFP accumulates in endomembrane vesicles in *scd1*-1, which is consistent with a role of SCD1 in controlling PIN endomembrane targeting. Auxin transport and endomembrane accumulation of a PIN2 reporter were examined both in *scd1-1* grown continuously at the restrictive temperature or in seedlings transitioned briefly to this temperature. The endomembrane bodies in which the PIN2 fusion protein is localized also accumulate other proteins that are linked to the late multivesicular endosome and the endosome dye FM4-64 after an extended incubation. These results provide support for the hypothesis that SCD1 protein functions to regulate the movement of PIN2 to and from the plasma membrane and that these transient changes in localization occur more rapidly than changes in auxin transport and dependent developmental processes.

## Results

### Root Elongation and Lateral Root Development Are Temperature-Sensitive in *scd1-1*

**FIGURE 1:**
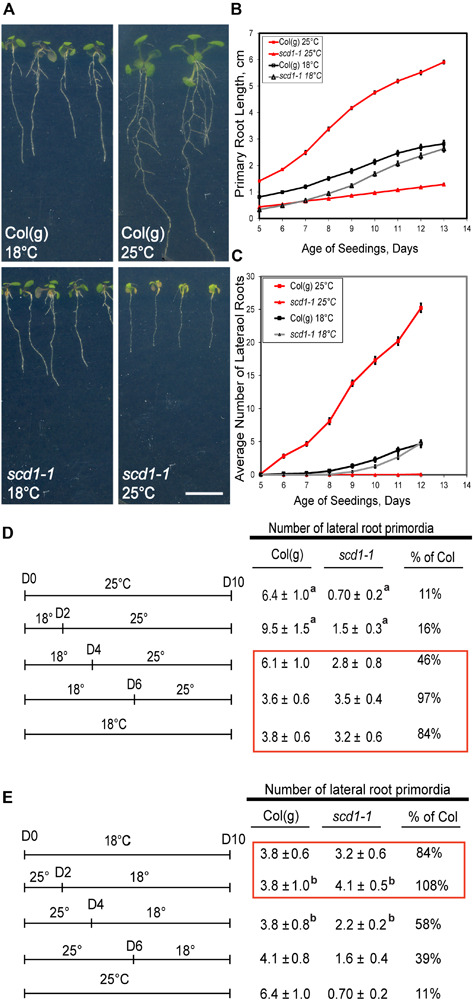
Root growth and lateral root formation in *scd1-1* are reduced at the restrictive temperature. **(A)** Col(g) and *scd1-1* roots 12 days after sowing grown under continuous light at 18°C or 25°C. **(B,C)** Primary root length **(B)** and lateral root number **(C)** were measured daily for Col(g) and *scd1-1* seedlings maintained at 25 or 18°C. The average and SE (*n* = 20) are reported, although some errors are smaller than the symbols. **(D,E)** For these experiments, the total number of lateral roots was determined at 10 days after germination, but seedlings were grown at the indicated temperatures prior to this quantification with the average and standard error reported for 20 seedlings. **(D)** Seedlings were grown continuously at 25°C, or at 18°C for the indicated number of days before transfer to 25°C, or continuously at 18°C, as indicated in the left-hand panel. In the right-hand panel, the number of lateral root primordia are reported. The red box indicates treatment when SCD1 function is sufficient for root formation. **(E)** The parallel experiment was performed where seedlings were grown continuously at either 18°C, or at 18°C and transferred to 25°C on the indicated day, or continuously at 25°C. The red box indicates treatment when SCD1 function is sufficient for root formation. ^a^Indicates significant differences in genotype between 18°C control and indicated treatment determined by Student’s *T*-test with *p* < 0.05. ^b^Indicates significant differences in genotype between 25°C control and indicated treatment as determined by Student’s *T*-test with *p* < 0.05.

If *scd1-1* has a defect in targeting of auxin transport proteins, then alterations in auxin transport-dependent root responses, including primary root elongation, gravitropism, and lateral root development, would be expected. Root growth and development of *scd1-1* were compared to its parental line, Columbia *glabrous-1* [(Col(g)], at 18°C and at 25°C. At 18°C, Col(g) and *scd1-1* had similar primary root elongation rates and lateral root numbers (Figure 1). At 25°C *scd1-1* primary roots were substantially shorter than Col(g) (Figure 1B), and the number of lateral roots were dramatically reduced (Figures 1B,C). We performed a two-way ANOVA to determine which of these differences were statistically significant across this time course. In both root elongation and lateral root development, *scd1-1* was significantly impaired relative to Col(g) at both temperatures with *p* < 0.01 for all comparisons.

The temperature sensitive phenotype of the *scd1-1* mutant allowed us to ask if shorter-term growth at 25°C could induce this lateral root phenotype and whether the impaired root development at this restrictive temperature could be rescued by transition back to the permissive temperature. Col(g) and *scd1-1* seedlings were grown at constant 18°C, or constant 25°C, or grown at 18°C and then transferred to 25°C after 2, 4, or 6 days (Figures 1D,E). For all these treatments, the number of lateral roots was quantified on the 10th day after sowing, there was a significant difference in the number of lateral root initiation events between 18 and 25°C for both Col(g) and *scd1-1*. Growth at 25°C increased lateral root formation in Col(g) and decreased lateral root formation in *scd1-1*. When *scd1-1* seedlings were grown at 18°C for 2 days before transfer to 25°C, significantly fewer lateral roots formed than in plants grown continuously at 18°C, while roots grown at 18°C for 4 or 6 days before transfer to 25°C formed an equivalent number of lateral roots to the continuous 18°C *scd1-1* control (red box, Figure 1D), consistent with SCD1 function at 18°C allowing lateral root development.

In a parallel experiment, Col(g) and *scd1-1* seedlings were grown at 25°C and transferred to 18°C after 2, 4, and 6 days (Figure 1E) to determine if the ability to form roots could be restored upon transition to the permissive temperature. There were significantly more lateral root primordia formed on *scd1-1* seedlings switched from 25 to 18°C at days 2 and 4 than seedlings grown at a constant 25°C or transitioned after 6 days. These two experiments indicate that there is a developmental window between 2 and 6 days in which SCD1 function is required for lateral root formation.

### Gravitropic Curvature Is Delayed in *scd1-1*

**FIGURE 2:**
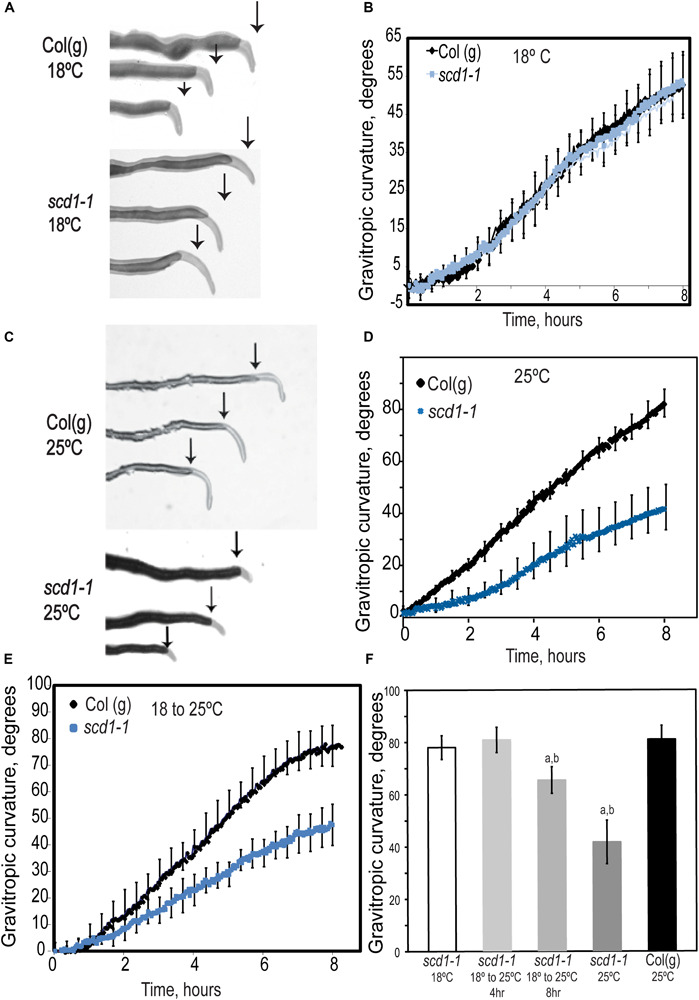
Gravitropic response of *scd1-1* at 25°C is impaired relative to wildtype. **(A,C)** Five day wildtype and *scd1-1* seedlings were grown at **(A)** 18°C or **(C)** 25°C then rotated 90° relative to the gravity vector, with images taken at up to 8 h after reorientation. The root tips of seedlings at 8 h after reorientation are overlaid on the image of the root before curvature and the arrow shows the position of the root tip at the time of reorientation. **(B,D)** Root curvature as a function of time after reorientation was quantified after reorientation for **(B)** roots grown at 18°C and **(D)** roots grown at 25°C. Error bars = ± SEM. **(E)** The gravitropic curvature of *scd1-1* and Col(g) plants grown for 5 days at 18°C then transferred to 25°C for 12 h prior to reorientation. **(F)** The gravitropic response of *scd1-1* was quantified in seedlings grown at 18°C and transferred to 25°C for 4 h or 8 h before being reoriented 90° relative to the gravity vector. Curvature was measured 8 h after reorientation. ^a^Denotes a significant difference in gravitropic curvature between *scd1-1* at the indicated treatment and *scd1-1* at 18°C and ^b^denotes a significant difference between treatment and Col(g) at 18°C. For all panels *n* = 12 seedlings of each genotype.

We examined the gravity response in wild type and *scd1-1* seedling roots. Wild type and *scd1-1* seedlings grown at 18 or 25°C for 5 days were rotated 90° (Figure 2). At 18°C, *scd1-1* and wild type had identical gravitropic responses. However, at 25°C, Col(g) showed a more rapid response than at 18°C, with root curvature reaching 80° and 50° by 8 h at 25° and 18°C, respectively. In contrast, *scd1-1* at 25°C showed a 50% reduction in the gravitropic response relative to Col(g), with root curvature reaching a mean angle of only 40°.

To eliminate developmental consequences of the *scd1-1* phenotype at the restrictive temperature on gravity response, we asked whether the gravity response would be impaired in plants grown at 18°C for 5 days and transferred to 25°C for 4–12 h prior to reorientation. The gravitropic response in the *scd1-1* mutant 12 h after transition to 25°C was slower than Col(g), impaired to levels equivalent to seedlings grown continuously at 25°C (Figure 2E). In contrast, *scd1-1* seedlings transferred from 18 to 25°C for 4 h before reorientation did not show a decrease in gravitropic curvature as compared to Col(g), while 8 h at 25°C led to a significant, but not complete, inhibition of curvature (Figure 2F). The elongation of roots of Col(g) and *scd1-1* seedlings grown at 18°C and then transferred to 25°C were also monitored (Supplementary Figure S1). A significant decrease in the growth rate of *scd1-1* was observed beginning at 5 h after transfer to 25°C, consistent with the impaired symmetric and asymmetric elongation of *scd1-1* roots at 25°C, but not 18°C.

It is possible that the gravity defect observed in *scd1-1* was due to a change in physical structure of the gravity perception machinery, either the columella cells or their statoliths. When roots were grown at 18°C or switched from 18 to 25°C for 24 h, Col(g) and *scd1-1* had similar numbers of statoliths per cell and number of columella cells, suggesting that the gravitropic defect in *scd1-1* was not due to altered perception of the gravity vector (Supplementary Figure S2).

### IAA Transport Is Impaired in the *scd1-1* Mutant at the Restrictive Temperature

**FIGURE 3:**
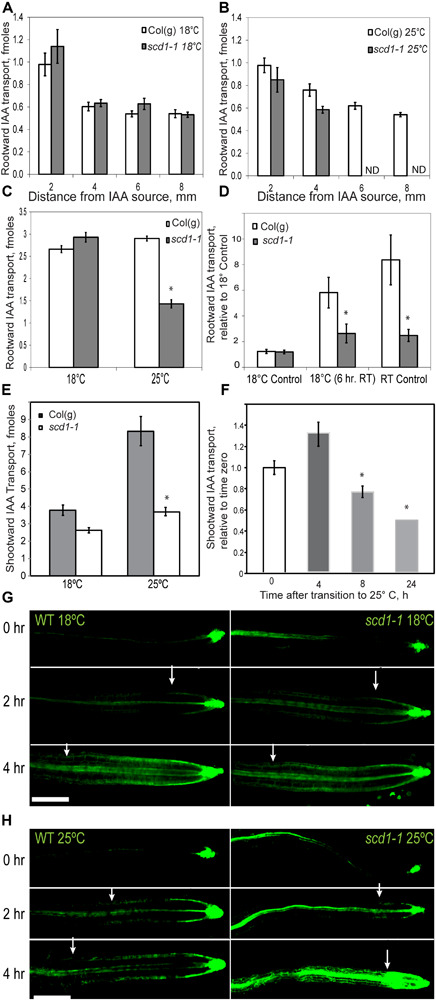
The *scd1-1* mutant has reduced IAA transport at the restrictive temperature. Rootward ^3^H-IAA transport was measured in a pulse chase assay using seedlings grown for 9 and 10 days for Col(g) and *scd1-1*, respectively, to match seedling length. Transported auxin was quantified in progressive 2 mm segments downstream of the site of application. For all panels, the averages and SE of 19–25 seedlings are reported. **(A)** Seedlings were grown at 18°C. **(B)** Seedlings were grown at 25°C. ND indicates that no radioactivity was detected. **(C)** Comparison of the total rootward IAA transport between Col(g) and *scd1-1* at 18 and 25°C, *p < 0.05. **(D)** Rootward IAA transport was determined for seedlings grown at constant 18 or 25°C or transitioned from 18 to 25°C for 6 h before assay. **(E)** Shootward IAA transport was measured in thirty 5 day-old seedlings grown at 18°C or 25°C. **(F)** *scd1-1* seedlings grown at 18°C were transferred to 25°C for 4, 8, and 24 h prior to the ^3^H-IAA transport assay. Significant reduction in IAA transport in *scd1-1* seedlings as compared to Col(g) is indicated *p < 0.05. **(G,H)** Col(g) and *scd1-1* seedlings expressing pDR5-GFP, were grown for 7 days and treated with unlabeled IAA at the root tip. Auxin-induced gene expression was reflected in the movement of fluorescence from the root tip toward the shoot in the epidermal tissue at the indicated times after application of IAA at the root tip at **(G)** 18°C and (H) 25°C. Arrowheads indicate the most distal position of epidermal GFP signal. Scale bar = 50 μm.

Rootward IAA transport, which is required for lateral root emergence (Reed et al., 1998; Bhalerao et al., 2002) was measured using an IAA pulse chase assay. At 18°C, rootward transport was equivalent in Col(g) and *scd1-1* (Figure 3A). At 25°C, the length of the zone of transported IAA was reduced (Figure 3B) and a significant reduction in the total rootward IAA transport (the sum of samples in Figures 3A,B) was evident in *scd1-1* relative to wild-type (Figure 3C). When rootward IAA transport in Col(g) and *scd1-1* grown at constant 18 or 25°C was compared to transport in plants grown at 18°C and transferred to 25°C for 6 h, reduced IAA transport was evident in *scd1-1* plants grown at constant 25°C or at 18°C and transferred to 25°C for 6 h (Figure 3D).

To determine whether shootward IAA transport, which controls gravity response (Rashotte et al., 2000), was impaired in *scd1-1*, ^3^H-IAA was applied to the root tip of 5-day old wild type and *scd1-1* seedlings grown at 18 or 25°C. After 2 h, shootward transport was measured. At 18°C, there was no significant difference between wild type and *scd1-1* in the amount of IAA transported (Figure 3E). At 25°C, wild type roots had a twofold greater shootward auxin transport compared to those grown at 18°C, while *scd1-1* showed equivalent levels of IAA transport at 18 and 25°C (Figure 3E). Thus unlike the wild type, *scd1-1* does not display a temperature-dependent increase in auxin transport, and had significantly less transport than Col(g) at 25°C. *scd1-1* seedlings were grown at 18°C for 5 days then transferred to 25°C, and the amount of shootward ^3^H-IAA transport was determined. Within 8 h of transfer there was a significant decrease in IAA transport compared to wild type, with a further decrease by 24 h (Figure 3F).

Wild type and *scd1-1* seedlings expressing the auxin-responsive promoter driven reporter (DR5-GFP) were used to follow movement of an auxin-induced gene expression signal using a previously published method (Lewis and Muday, 2009). Unlabeled IAA was applied to the root tips of wild type and *scd1-1* seedlings, and the spread of GFP fluorescence in epidermal cells from the root tip toward the shoot was visualized (Figures 3G,H). Before application of IAA, epidermal fluorescence was limited to the root tip (Figure 3G). At 18°C, for up to 4 h after IAA application, a similar steady elongation of the zone of fluorescence along the root tip was observed in wild type and *scd1-1* seedlings (Figure 3G). However, at 25°C, *scd1-1* roots showed a significantly slower shootward progression of GFP signal than wild type, and even accumulated a bolus of fluorescence near the root tip, consistent with the ability to take up IAA, but not to transport IAA away from the root tip (Figure 3H). These results are consistent with impaired IAA transport from the root tip at the restrictive temperature leading to reduced growth and gravitropic curvature.

### Gravity-Directed Asymmetric pDR5-GFP Expression Is Altered in *scd1-1*

**FIGURE 4:**
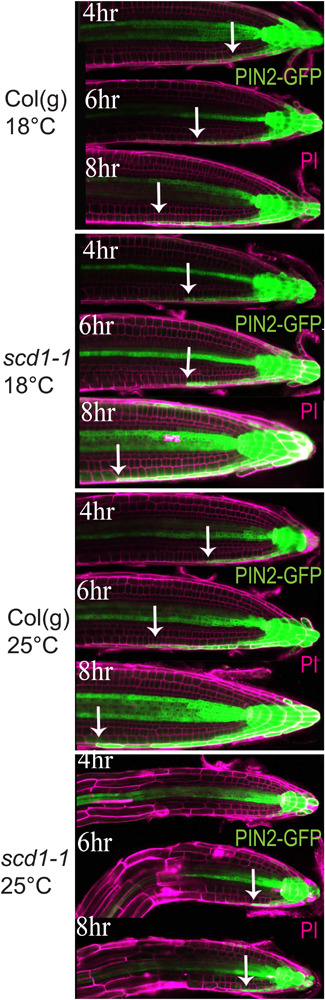
*scd1-1* shows decreased lateral distribution of auxin at 25°C as compared to wild type. Col(g) and *scd1-1* pDR5-GFP-expressing seedlings were grown vertically for 5 days at 18 or 25°C. After 90° rotation, the asymmetrical pDR5-GFP expression was observed at the root tip at 4, 6, and 8 h after reorientation. The arrows indicate the position farthest from the root tip where asymmetric GFP fluorescence was detected. The seedlings were stained with propidium iodide (magenta) to delineate the cell walls. Representative images from more than 5 roots of each genotype and time point are shown. Scale bar = 50 μm.

Wild type and *scd1-1* seedlings containing a reporter of auxin-induced gene expression in which the auxin-responsive DR5 artificial promoter drives GFP synthesis (DR5-GFP) were used to determine whether the altered gravitropic response of *scd1-1* was accompanied by a temperature-sensitive defect in the lateral redistribution of auxin at the root tip. Seedlings were grown at 18°C for 5 days then were reoriented 90°, and then imaged at 2 h intervals after staining with propidium iodide (PI) to reveal the boundaries of cells. GFP fluorescence was captured in optical slices through the center of the root with settings optimized to visualize the signal in the epidermis on the lower side of the root. At 18°C roots of both genotypes showed asymmetric auxin-induced GFP fluorescence signal with the GFP signal moving a greater distance from the tip back on the lower side of roots beginning 4 h after reorientation (Figure 4). At 25°C, the wild type seedlings showed this same asymmetric GFP fluorescence, also beginning at 4 h post-reorientation. However, in *scd1-1* at 25°C, weak asymmetric DR5-GFP fluorescence was not detected until 6 h post-re-orientation and was substantially less than *scd1-1* at 18°C and wild type at either temperature. These data are consistent with reduced asymmetric auxin transport from the root tip affecting the shootward progression of auxin in *scd1-1* roots reoriented relative to the gravity vector at 25°C, consistent with a role of SCD1 protein in auxin transport-dependent processes.

### *scd1-1* Shows Altered Localization of PIN Proteins

**FIGURE 5:**
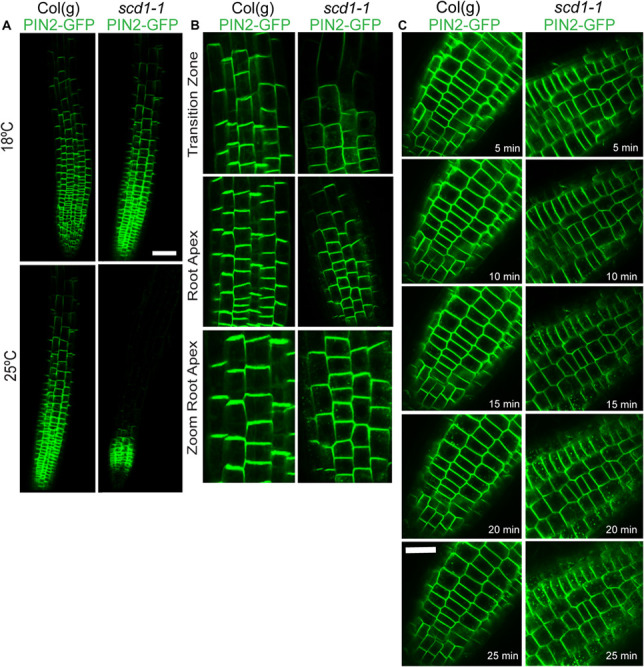
PIN2-GFP shows altered localization in the root tips of the *scd1-1* mutant after constitutive growth or transition to the restrictive temperature. **(A)** Wild type and *scd1-1* seedlings were grown at 18 and 25°C. Scale bar = 50 μm **(B)** Higher magnification images of Col(g) and *scd1-1* seedlings grown for 7 days at 25°C. Scale bar = 20 μm. **(C)** Roots were grown at 18°C for 7 days then transferred to 25°C for the indicated times after transfer. Images representative of more than 100 roots are shown. Scale bar is 20 μm.

The effects of the *scd1-1* mutation on root development, gravitropism, and IAA transport suggests altered targeting of auxin transport proteins. To test this hypothesis, the distribution of *PIN2pro::PIN2-*GFP (PIN2-GFP) fusion protein was examined in wild type and *scd1-1* seedlings (Figure 5). Wild type seedlings grown at 18 and 25°C, and *scd1-1* seedlings grown at 18°C displayed a typical asymmetric plasma membrane localization of PIN2-GFP at the shootward face of the plasma membrane of root epidermal cells. The *scd1-1* seedlings grown at 25°C showed a reduced region of plasma membrane-localized PIN2-GFP fluorescence, with expression limited to epidermal cells near the root tip, disappearing at the start of the maturation zone (Figure 5A). *scd1-1* seedlings grown at 25°C also revealed PIN2-GFP signal in endomembrane bodies, aberrant vesicular compartments in epidermal and cortical cells, similar to those reported previously in response to treatment with chemical that blocks targeting (Drakakaki et al., 2011). The PIN2-GFP endomembrane accumulation was most profound and consistent in the cortical cells in the transition zone (Figure 5B).

To determine the time course of the appearance of the PIN2-GFP containing endomembrane bodies in the root epidermis, *scd1-1* seedlings were grown at 18°C, shifted to 25°C, and observed continuously for 35 min on a temperature-controlled stage at 25°C. In *scd1-1*, the PIN2-GFP signal was detected in endomembrane bodies 10–15 min after temperature shift, with an increase in frequency continuing over the next 10–15 min (Figure 5C). In Col(g), PIN2-GFP was rarely detected in endomembrane bodies, although a few are evident at 20 min after transition to 25°C.

**FIGURE 6:**
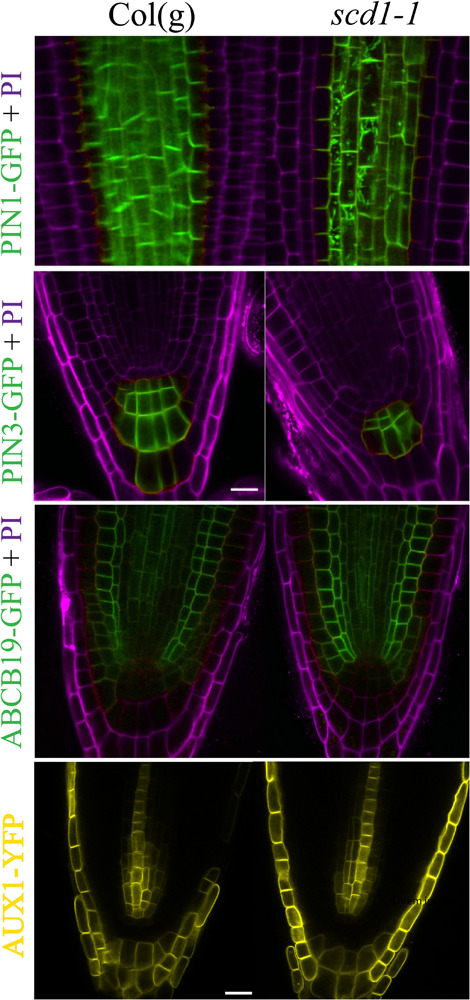
PIN1, but not PIN3, ABCB19, or AUX1, was found in endomembrane bodies in *scd1-1* mutants. Col(g) and *scd1-1* seedlings expressing PIN1-GFP, PIN3-GFP, ABCB19-GFP (green), and AUX1-YFP (yellow) were grown for 7 days and imaged by LSCM using identical confocal settings after growth at the non-permissive temperature of 25°C. Propidium iodide was used to stain the cell wall (magenta). Representative images from at least 5 roots are shown. Scale bar = 10 μm.

PIN1 localizes to the rootward surface of cells in the root vascular cylinder (Galweiler et al., 1998). The *PIN1pro::PIN1-*GFP(PIN1-GFP) was also found in endomembrane bodies in *scd1-1* mutants at 25°C, but not in wild type (Figure 6). However, at 25°C, *PIN3pro::PIN3-*GFP (PIN3-GFP) was not detected in endomembrane bodies. The localization of ABCB19 and AUX1 fused to fluorescent proteins was not altered in *scd1-1* (Figure 6). These data suggest that without fully functional SCD1 protein, the membrane targeting of PIN1 and PIN2 auxin transport proteins is disrupted.

### *scd1-1* PIN2-GFP Endomembrane Bodies Co-localize With FM4-64

**FIGURE 7:**
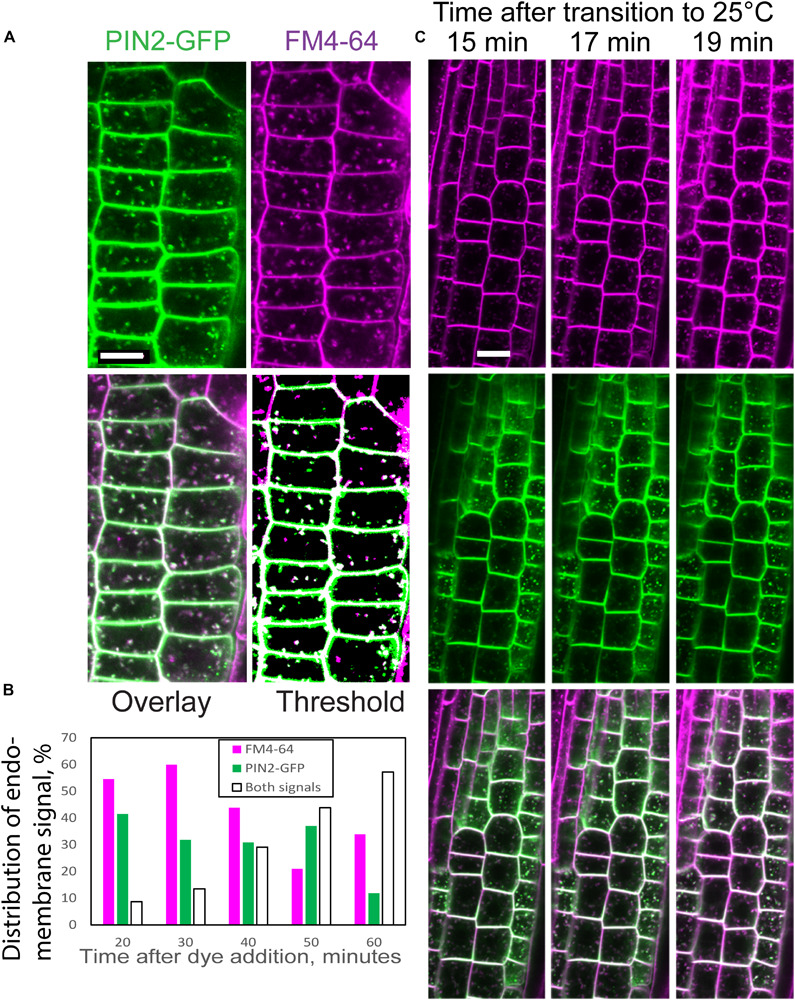
Colocalization of PIN2-GFP and FM4-64 in *scd1-1*. **(A)** An *scd1-1* root containing PIN2-GFP (green) incubated in FM4-64 (magenta) for 20 min, rinsed, and imaged after an additional 40 min (a total of 60 min after initiation of staining). Colocalization is indicated in the overlay as white. The merged image was enhanced using a pseudocolor fill of a thresholded image (last panel). Scale bar = 10 μm. **(B)** Roots were stained in FM4-64 for 20 min, transferred to water, and imaged immediately (20 min) or at later times as indicated. The percentage of endomembrane bodies containing GFP, FM4-64, or both signals are plotted as a function of time after initiation of staining with FM4-64. The total number of roots examined was 21. At 20 min: *n* = 3, at 30 min: *n* = 5, at 40 min: *n* = 5, at 50 min: *n* = 4, at 60 min: *n* = 4. **(C)** The time course of colocalization was examined in *scd1-1* PIN2-GFP roots shifted from 18 to 25°C. *scd1-1* seedlings grown at 18°C for 7 days were transferred to 5 μM FM4-64 for 5 min, rinsed briefly, and then imaged by confocal microscopy at the indicated intervals on a temperature-controlled stage at 25°C. The PIN2-GFP (green) and FM4-64 (magenta) channels are shown separately and in the overlay, where the overlap of both signals is shown in white. Scale bar is 10 μm.

We used the endocytic tracer FM4-64 (Bolte et al., 2004) to determine whether the PIN2-containing endomembrane bodies in *scd1-1* at 25°C were endosomes. Root tips of PIN2-GFP *scd1-1* were incubated in FM4-64 for 20 min and co-localization of the fluorescent signals was quantified over time. Images captured at 60 min after treatment with FM4-64 contain endomembranes that exhibit FM4-64 fluorescence (magenta) or PIN2-GFP fluorescence (green), with the majority of endomembrane bodies containing both fluorescent signals (Figure 7A). These images contrast with *scd1-1* roots treated with FM4-64 for 20 min, where the endomembrane bodies had either PIN2-GFP fluorescence or FM4-64 signal (Supplementary Figure S3) and with Col(g) root where no PIN2-GFP was visible in endomembranes, and there was no colocalization of FM4-64 and PIN2-GFP signal (Supplementary Figure S4). A threshold function was applied to individual channels to quantify co-localization independent of intensity in more than 30 cells from multiple roots at each time point. The thresholded overlay in which pixels were indicated as magenta for FM4-64, green for PIN2-GFP, and white for overlay of FM4-64 and GFP is shown in Figure 7A and Supplementary Figure S3. The percentage of the endomembrane bodies that showed overlapping signals increased steadily over the 60 min time course (Figure 7B), consistent with GFP accumulating in the late endosomes in *scd1-1*.

To provide details of the kinetics of this response, the accumulation of FM4-64 and PIN2-GFP was observed over time in root tips of *scd1-1* seedlings grown at 18°C for 7 days and placed in FM4-64 for 5 min before transition to 25°C. The formation of PIN2-GFP-positive endomembrane bodies was observed within 15 min after transfer to the non-permissive temperature and remained at relatively constant levels over a 30 min window (Figure 7C). The frequency and brightness of FM4-64 stained endomembranes increases as the dye moves from early to late endosomes. At 15 min some colocalization of FM4-64 and PIN2-GFP was observed, which increased over time with almost complete overlapping endomembrane signals by 20 min. The non-overlapping signals for PIN2-GFP and FM4-64 at early time points and increased colocalization at later time points is consistent with PIN2-GFP accumulation in late endosomes.

### *scd1-1* PIN2-GFP Endomembrane Bodies Co-localize With ARA7 and ARA6

**FIGURE 8:**
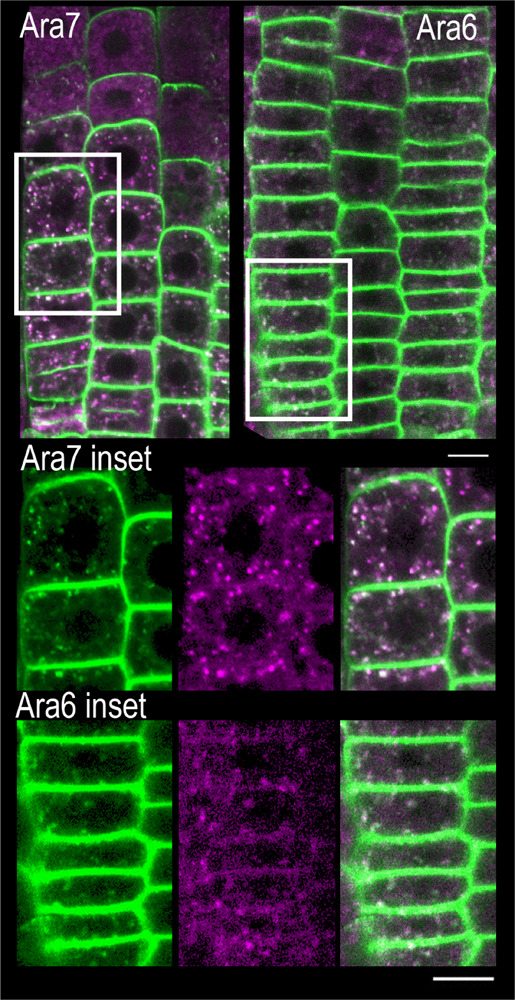
PIN2 overlays ARA7 and ARA6 in endomembrane bodies found in the root tips of *scd1-1* mutants at 25°C. RFP-ARA7 or ARA6-RFP (magenta) were crossed into *scd1-1* containing PIN2-GFP (green) roots and fluorescence visualized by confocal microscopy in 7 day old seedlings. Pixels with both green and magenta fluorescence are shown in white. When *scd1-1* seedlings expressing PIN2-GFP and RFP-ARA7 were grown at 25°C, endomembrane bodies were observed that have only PIN2-GFP signal (green) or RFP-ARA7 (magenta) signal, while a subset contain both signals (white). The insets are higher magnifications of the areas delineated by the white boxes above. For each reporter line, more than 20 seedlings were examined. The scale bar is 10 μm.

**FIGURE 9:**
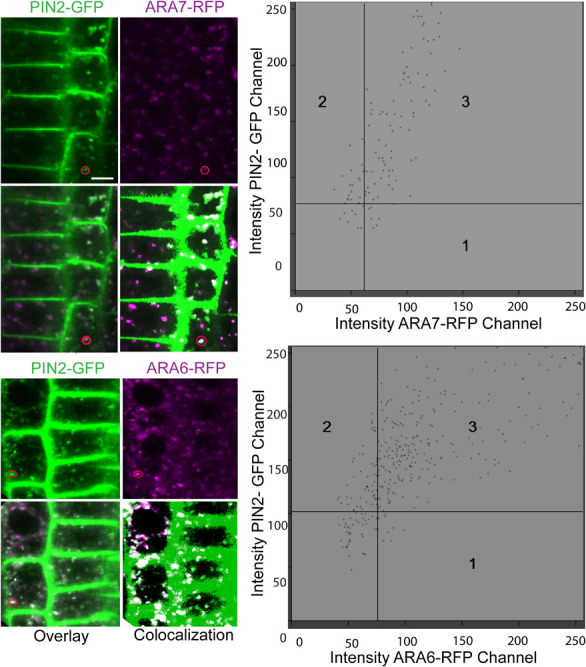
PIN2 co-localizes with ARA7 and ARA6 in endomembrane bodies *scd1-1* mutants at 25°C. Roots of *scd1-1* containing ARA7-RFP or ARA6-RFP (magenta) and PIN2-GFP (green) roots were visualized by confocal microscopy in 7 day old seedlings. The overlay shows pixels with both green and magenta fluorescence as white. Using colocalization software, thresholds were generated to better illustrate pixels with both fluorescent signals from 4 roots of each line. Colocalization graphs are shown for endomembrane bodies that are circled.

To identify the specific endomembrane compartment in which PIN2-GFP accumulated in *scd1-1* mutants grown at 25°C, these lines were crossed with plants expressing red or cyan fluorescent protein fusion markers for five distinct endomembrane compartments (Supplementary Table S1). ARA6 and ARA7 are RAB5 GTPase homologs, found in recycling endosomes (ARA6) (Ebine et al., 2012) or in late endosomes, prevacuolar compartments and the trans Golgi network (ARA7) (Lee et al., 2004; Haas et al., 2007; Jia et al., 2013; Cui et al., 2014; Ito et al., 2018). When *scd1-1* seedlings expressing PIN2-GFP (green) and RFP-ARA7 (magenta) were grown at 25°C, endomembrane bodies were observed that have only PIN2-GFP signal (green) or RFP-ARA7 (magenta) signal, while a subset contain both signals (white) (Figure 8). Higher magnification images of this localization are shown in Figure 9. To determine if PIN2-GFP in endomembrane bodies colocalized with both of these markers in *scd1-1* mutants we performed a quantitative colocalization analysis examining signal in multiple PIN2-GFP positive endomembranes. The weighted Manders coefficients (Manders et al., 1993) for the colocalization of PIN2-GFP were 0.94 and 0.91 with ARA7 and ARA6, respectively, and Pearson’s overlap coefficients (Dunn et al., 2011) were 0.82 and 0.83, respectively (Supplementary Table S2). The presence of both RFP-ARA7 or ARA6-RFP and PIN2-GFP in a subset of these vesicles is consistent with other cargo proteins that move through a subset of the ARA7-positive endosomes interacting with distinct sets of RAB5 proteins (Dunn et al., 2011; Ito et al., 2018).

We found little or no overlap in the endomembrane localization of PIN2-GFP fluorescence in lines containing reporters localized in either the early endosome (VHA1-RFP) (Geldner et al., 2007) TGN (SYP61-CFP) (Robert et al., 2008), or the sorting endosome (SNX1-RFP) (Jaillais et al., 2006, 2007; Supplementary Figure S5). These results are consistent with PIN2-GFP in *scd1-1* accumulating in late multivesicular endosomes and not in other compartments.

## Discussion

Polar transport of the hormone auxin from cell to cell is controlled by the asymmetric localization of the PIN family of IAA efflux carriers (Grunewald and Friml, 2010; Zazimalova et al., 2010; Adamowski and Friml, 2015). These carrier proteins are directed to specific plasma membrane domains through dynamically regulated membrane trafficking and recycling pathways (Geldner, 2009; Grunewald and Friml, 2010; Barbosa and Schwechheimer, 2014). Internal and external environmental cues also affect the localization of the PIN proteins through regulation of the flow of PIN proteins between the endosome and plasma membrane (Paciorek et al., 2005; Jasik et al., 2013, 2016; Rakusova et al., 2015), as well as between the plasma membrane and the lytic vacuole (Kleine-Vehn et al., 2008a).

In this study we utilized a temperature-sensitive *scd1-1* mutant to uncover the dynamics of auxin transport targeting and how these reversible changes in protein targeting control auxin transport and dependent process. SCD1 contains a DENN domain found in proteins with RAB-directed GEF activity and in proteins that interact with clathrin (Patino-Lopez et al., 2008; Sato et al., 2008; McMichael et al., 2013). *SCD1* null mutants have revealed the function of this protein as a RAB-GEF that participates in both endocytosis and exocytosis (Mayers et al., 2017).

The effects of the temperature-sensitive *scd1-1* mutant on root development was examined at the permissive and restrictive temperatures of 18 and 25°C, respectively. We found that *scd1-1* exhibits a suite of phenotypes only at restrictive temperature, which include impaired root elongation and gravity response, reduced lateral root formation, and inhibition of formation of asymmetric auxin-induced gene expression across a gravity-stimulated root. These phenotypes were also induced when plants were grown at the permissive temperature and then transitioned to the restrictive temperature and could be reversed when plants were transitioned from the restrictive temperature back to the permissive temperature. As all of these phenotypes are dependent on auxin transport, we also measured both shootward and rootward transport and found that both are impaired at the restrictive temperature.

The ability to reversibly manipulate lateral root development in the *scd1-1* mutant provided unique insight into how this process is regulated and revealed the critical time point when SCD1 and auxin transport are needed for lateral root development. A temperature-switch assay was performed resulting in the shift of seedlings from the permissive temperature to the non-permissive temperature or vice versa over 10 days. These data support the hypothesis that SCD1 is needed for lateral root development between days 2 and 6. Transition from the permissive to non-permissive temperature also reduces auxin transport in *scd1-1* (but not Col(g)) showing reductions in the long distance rootward transport within 8 h after transition. A previous report suggested that there is specific regulation of auxin transport in this time window with a pulse of auxin moving from the shoot to the root at 3–5 days, which correlated with the emergence of the first lateral root (Bhalerao et al., 2002). Our ability to reversibly alter auxin transport by turning off or on SCD1 function support the model of an auxin pulse that drives root formation that occurs within a precise developmental window.

The effects of the *scd1-1* mutation on auxin transport and gravitropism were evident after both long and short-term exposure to the non-permissive temperature, with changes in auxin transport evident preceding gravitropic differences. When grown continuously at 25°C or within 8 h after transfer from the permissive 18°C temperature, *scd1-1* seedlings showed reduced shootward ^3^H-IAA transport and accumulation of auxin-induced gene expression at the root tip, consistent with impaired auxin transport away from the site of application. Similarly, continuous growth at 25°C or a shift from 18 to 25°C for 8 h showed a significant decrease in the gravitropic curvature in *scd1-1* compared to wild type. Auxin-induced gene expression, visualized via DR5-GFP fluorescence (Muday and Rahman, 2008; Geisler et al., 2014) was rapidly enhanced on the lower side of Col(g) roots after gravitropic reorientation, but was impaired in *scd1-1* seedlings relative to wild type only in plants grown at 25°C. These data support the hypothesis that a functional SCD1 protein is needed to maintain polar and lateral auxin transport and the physiological processes dependent upon dynamic changes in appropriate auxin distribution. Consistent with this model, altered PIN2-GFP trafficking is even more rapidly perturbed in *scd1-1* than auxin movements and gravity response.

Because PIN2 is required for rootward transport and root gravitropism (Muller et al., 1998; Rashotte et al., 2000; Chen et al., 2002), we compared its localization in the *scd1-1* mutant and wild type using a PIN2-GFP reporter. At 25°C, PIN2 is normally localized to the plasma membrane on the shootward end of cells of the lateral root cap and epidermis of the developing root of wild type seedlings (Muller et al., 1998). In the *scd1-1* mutant, PIN2-GFP was not restricted to the plasma membrane but was also localized in endomembrane bodies. Mayers et al. (2017) showed that *scd1-2* has altered PIN2-GFP trafficking during treatment with brefeldin A, a drug which blocks intracellular endosome and trans-Golgi transport. However, we find that in *scd1-1* at the restrictive temperature, PIN2-GFP accumulates in the absence of BFA.

The trafficking defect is not detectable in *scd1-1* roots grown at 18°C, but when switched from 18 to 25°C, the PIN2 trafficking defect becomes apparent within minutes. Since changes in auxin transport and root growth and development are not observed for 8–12 h, this suggests that these responses are sequential and that PIN mislocalization is not a secondary result of compromised root development at 25°C. The linkage is most evident in terms of gravity response. PIN2 controls shootward auxin transport that is required for gravitropism (Muller et al., 1998; Rashotte et al., 2000; Chen et al., 2002). Upon transition of *scd1-1* to the non-permissive temperature, PIN2-GFP endomembrane accumulation is evident with less than hour. In Col(g) a transition from 18 to 25°C increases shootward transport by more than fivefold within 5 h, leading to a more than twofold greater transport in Col(g) than in *scd1-1*. Treatment of roots for 12 h at the non-permissive temperature impaired gravity response in *scd1-1* to levels consistent with constitutive growth at this non-permissive temperature. Together these results support the timeline where PIN2 targeting is impaired, reducing auxin transport and then dependent physiological processes.

We also detected l PIN1-GFP within endomembrane bodies in *scd1-1*. However, other fluorescent fusions to the auxin transport proteins PIN3, ABCB19, and AUX1, were not observed in any endomembrane compartments. As all of these proteins are trafficked and recycled, and the *scd1-1* mutation most strongly affects PIN1 and PIN2 and dependent physiological processes, there appears to be selectivity of SCD1 targets toward PIN2 and PIN1, which is consistent with other studies that reveal distinct regulatory and trafficking machinery between auxin transporters (Barbosa and Schwechheimer, 2014; Weller et al., 2017). Other reports have indicated that ABCB19 and AUX1 localization are regulated by different trafficking mechanisms than those that regulate PIN1 and PIN2 (Kleine-Vehn et al., 2006; Titapiwatanakun et al., 2008). The results are based on differences in the trafficking of these proteins in response to brefeldin A and FM4-64 uptake (Titapiwatanakun et al., 2008).

We find that in *scd1-1* at 25°C, PIN2-GFP accumulates in the late or multivesicular endosome. Impaired uptake of the endosomal marker dye FM4-64 has previously been observed in *scd1-1*, suggesting that SCD1 participates in endocytosis via clathrin coated vesicles (McMichael et al., 2013). To determine whether FM4-64 accumulates in the PIN2 endomembrane bodies observed here, the roots of *scd1-1* containing PIN2-GFP grown at 18°C and transferred to 25°C were treated with FM4-64. Although initially there was little co-localization, within 20 min significant overlap was observed. These results are consistent with PIN2 being trapped in *scd1* mutants in a late or multivesicular endosome or pre-vacuolar compartment on its way to or from the plasma membrane through the endosome.

To confirm that the endomembranes in which PIN2-GFP accumulates in *scd1-1* are late endosomes, we crossed multiple endosomal markers into this mutant and performed colocalization analyses. In *scd1-1* at 25°C, but not 18°C, we observed PIN2-GFP colocalization with ARA7 and to a lesser extent with ARA6. ARA6 and ARA7 are RAB5 GTPases contained in late multivesicular endosomes, with related, but not identical trafficking activities (Ueda et al., 2004; Beck et al., 2012; Ebine et al., 2012; Paez Valencia et al., 2016). ARA7 function is required for vesicle transport between the pre-vacuolar compartment (PVC) and the vacuole (Lee et al., 2004; Haas et al., 2007; Jia et al., 2013) and ARA6 is associated with transport between the PVC and the plasma membrane, where it may regulate trafficking from the endosome to the plasma membrane (Ebine et al., 2011, 2012). PIN2 has also been localized to ARA7 vesicles in *Arabidopsis* roots after treatment with endosidin 16, which inhibits apical membrane trafficking (Li et al., 2017).

The colocalization of PIN2 with both ARA7 and ARA6 Rab-related GTPases is not surprising considering that compartments bearing ARA7 and ARA6 have been previously shown to partially overlap, despite evidence that they are distinct endosomal compartments (Ueda et al., 2004; Beck et al., 2012; Ebine et al., 2012). The PVC, where both ARA6 and ARA7 are found, is a distinct organelle in plants that facilitates the transport of materials from the endosomes to the vacuole (Contento and Bassham, 2012). Using immunogold and confocal microscopy, plasma membrane proteins that pass through the PVC have been shown to be directed to the vacuole for degradation (Haas et al., 2007; Viotti et al., 2010). Thus, association of PIN2 with the PVC in *scd1-1* suggests that PIN2 in the PVC is likely targeted to the vacuole for degradation. This could explain the reduced domain of PIN2-GFP staining seen in the mutants at the restrictive temperature, coincident with the changes in columella cells. However, there is also evidence that suggests that PIN2 can be retrieved from the PVC and recycled (Kleine-Vehn et al., 2008b; Spitzer et al., 2009). Indeed, PIN1, PIN2, and AUX1 are targeted to the vacuole through the multi-vesicular body (MVB), and RabF2A co-localizes in the MVB with PIN1-GFP (Spitzer et al., 2009).

The localization of PIN2-GFP in late endosomal compartments in *scd1-1* is consistent with reports that PIN protein turnover involves both cycling between the endosome and plasma membrane and vacuolar degradation, depending on environmental conditions (Paciorek et al., 2005; Kleine-Vehn et al., 2008b; Grunewald and Friml, 2010; Jasik et al., 2013). For example, when roots are reoriented relative to the gravity vector, enhanced endocytosis of PIN2 at the apical membrane followed by degradation through sorting to a proteolytic endosomal compartment resulted in asymmetric distribution of PIN2, redirected auxin flow, and root bending (Abas et al., 2006). The mis-localization and loss of membrane PIN2 in *scd1-1* explains the observed reductions in auxin transport and the observed phenotypes.

The efficient targeting of auxin transport proteins to the plasma membrane is required for appropriate auxin transport and dependent physiological processes. In this paper we have shown that the conditional *scd1-1* mutant, with a defect in a gene that functions as a RAB-GEF (Mayers et al., 2017), has altered localization of PIN2, with accumulation of this protein in endomembrane bodies. The PIN2-GFP containing endomembrane bodies in *scd1-1* showed dye accumulation and fluorescent protein reporter expression consistent with a late endosomal/prevacuolar character. Consistent with this altered protein localization, polar auxin transport and gravity response are impaired in *scd1-1*. The kinetics of the alterations in these responses upon transition to the non-permissive temperature demonstrate that altered trafficking of PIN2 is observed within minutes of transition to this temperature, while auxin transport and gravity defects are measured hours later, and root developmental defects can be quantified days after transition. These findings provide direct evidence that disruption of the targeting machinery which disrupts PIN2 protein localization precedes reductions in auxin transport and changes in auxin transport-dependent physiological processes.

## Materials and Methods

### Growth of Mutant and Transgenic Seedlings

Seeds were sterilized (75% ethanol, 0.01% Triton X-100) for 2 min, followed by a 95% ethanol rinse. The culture medium was 0.8% (w/v) agar (MP Biomedical), 1X MS nutrients (macro and micro salts: MSP01-50LT, Caisson Labs, Inc.), vitamins (1 μg/mL^–1^ thiamine, 1 μg/mL^–1^ pyridoxine HCL, and 0.5 μg/mL^–1^ nicotinic acid), 1.5% sucrose (w/v), and 0.05% MES (w/v); pH adjusted to 6.0 with 1M KOH. The seeds were placed in the dark at 4°C for 48 h before being moved to the permissive (18°C) or the restrictive (25°C) temperatures. The seedlings were grown vertically under continuous light at an intensity of 100 μmol m^–2^s^–1^ for 5 or 7 days at the specified temperature.

The Arabidopsis *scd1-1* mutant (Falbel et al., 2003) was crossed with a number of fluorescent protein fusions all driven by their endogenous promoters: PIN2-GFP (Xu and Scheres, 2005), PIN1-GFP (Heisler et al., 2005), PIN3-GFP (Laskowski et al., 2008), MDR1(ABCB19)-GFP (Wu et al., 2007), AUX1-YFP (Swarup et al., 2008), and the auxin responsive promoter driven GFP reporter, DR5-GFP (Ottenschläger et al., 2003; Lewis et al., 2011). The F2 generation was screened for lines homozygous for mutant phenotype and for reporter GFP expression. *scd1-1* containing PIN2-GFP was also crossed with reporters that localize to specific endomembrane compartments, with these organelle specific reporters listed in Supplementary Table S1.

### Characterization of *scd1-1* Root Length and Lateral Root Development

Seedlings of similar size were placed on fresh agar plates on the 5th day after growth at 18 or 25°C. Lateral roots initiation events corresponding to stages VI and VII of primordia (Malamy and Benfey, 1997) and emerged lateral roots (defined as any root that broke through the epidermis of the primary root) were quantified using a dissecting microscope.

To examine lateral root formation in seedlings that were moved between temperature conditions, Col(g) and *scd1-1* seedlings were grown at 18 or 25°C continuously or transferred from 18 to 25°C at 2, 4, or 6 days. Parallel experiments were performed where seedlings were grown at 25°C continuously or transferred from 25 to 18°C at the indicated times. The number of lateral root initiation events and the number of emerged lateral roots were quantified by DIC microscopy using the Zeiss AxioObserver inverted microscope with a Plan Apochromatic 40x objective and the reported data is from 20 seedlings.

### IAA Transport Assays

Pulse-chase rootward (acropetal) IAA transport assays were performed using a previously published method (Lewis and Muday, 2009) in Col(g) seedlings at 9 and 6 days at 18 and 25° C, respectively. *scd1-1* seedlings were assayed at 10 days for both 18°C and 25°C to more closely match the length of *scd1-1* and Col(g). An agar droplet (1.25% agar w/v) containing 1 μM tritiated IAA was placed at the root shoot junction and incubated at 18 or 25°C for 10 min. The seedlings were transferred to a new agar plate and exposed to a “chase” droplet of unlabeled 1 μM IAA. After 50 min, 2 mm sections were excised beginning at the base of the droplet toward the root tip. Three to six segments were recovered from all plants, and each segment was counted separately, and IAA levels quantitated using a Beckman LS6500 liquid scintillation counter. The average and SE of 19–25 seedlings is reported in all transport assays.

Shootward IAA transport was measured by applying 500 nM tritiated IAA (^3^H-IAA) to root tips (Lewis and Muday, 2009) of 5 day seedlings grown at 18 or 25°C. For temperature switch experiments, seedlings were transferred to the appropriate temperature 4, 8, and 24 h before assay. Agar droplets containing 500 nM ^3^H-IAA were applied to the root tips, and seedlings were placed into the indicated temperatures under yellow filters to prevent auxin degradation (Stasinopoulos and Hangarter, 1990). After 2 h, the droplets were removed from the root tip. A 5 mm segment was excised 2 mm from the site of ^3^H-IAA application, and radioactivity was measured.

Shootward IAA transport was also measured indirectly using the artificial DR5 promoter driving expression of green fluorescent protein (GFP) (Ottenschläger et al., 2003). Droplets containing 1 μM unlabeled IAA in 1.25% agar were applied to the root tips of 5-day old seedlings grown at 18 or 25°C for the designated times. GFP fluorescence was visualized using a Zeiss 710 LSCM (Jena, Germany) using the argon laser with excitation at 488 nm. Images of root tips were captured using 80% laser power with 40x magnification, a pinhole of 2.64 airy units (AU), 560 gain and an emission spectrum of 493-656 nm. Images were exported to Adobe Photoshop, and the distance of GFP fluorescence in the epidermal layers was measured using the line tool.

### Gravitropic Response Assays

Five-day old Col(g) and *scd1-1* seedlings grown at 18°C were placed in diffuse light at 20–22 μmol m^–2^ s^–1^ and re-oriented 90°. Images were captured for low temporal resolution analysis at time 0 and at 2 h intervals until 8 h after re-orientation, with a final image 24 h after re-orientation. Images were captured using an Epson Perfection 3200 Photo Perfection flatbed scanner (Nagano, Japan) with 16-bit grayscale and 800 dpi resolution. Images were exported to ImageJ to measure degree of curvature.

The gravitropic root response was also observed using high temporal resolution imaging using an AVT Marlin camera (Stadtroda, Germany). Root curvature after gravity stimulation was measured by a MATLAB based custom image analysis software (Lewis et al., 2007) and is the average and standard error of 12 seedlings of each genotype.

### Asymmetric Auxin Induced Gene Expression After Gravitropic Reorientation

Col(g) and *scd1-1* seedlings crossed with the proDR5-GFP construct were imaged after roots were reoriented 90° relative to the gravity vector. Seedlings were placed on agar plates on the 5th day after growth at 18 or 25°C. After 1 h, seedlings were placed in diffuse light at 20–22 μmol m^–2^s^–1^ and reoriented 90°. Images were captured at time 0 and at 2 h intervals until 8 h after re-orientation. Seedlings were stained with propidium iodide (PI) to visualize root structure and mounted in dH_2_0 on slides. The root tips were visualized with the 40x objective of a Zeiss 710 LSCM using the argon laser at an excitation wavelength of 488nm and a pinhole of 1.0 AU. GFP emission was collected between 492 and 547 nm, while PI was collected at 567–735. All images were collected at identical gain and laser power and exported to Adobe Photoshop for analysis. Representative images of more than 5 roots at each time point and from each genotype were shown.

### Localization of Auxin Transport Protein-Fluorescent Protein Fusions

Col(g) and *scd1-1* seedlings containing PIN2-GFP, PIN1-GFP, PIN3-GFP, ABCB19-GFP, and AUX1-YFP (protein fusions driven by endogenous promoters) were grown for 7 days at a constant 18 or 25°C. Seedlings were mounted in dH_2_O and maintained at the indicated temperatures using a Julabo F25 temperature stage (Seelbach, Germany). Images were captured using a 63x water objective with or without a 1.5x optical zoom on a Zeiss 710 LSCM, using the argon laser for excitation at 488 nm with a pinhole of 1.0AU. PIN2-GFP, PIN1-GFP, PIN3-GFP, and MDR1-GFP were imaged with an emission spectrum of 500–525 nm, while AUX1-YFP was imaged with an emission spectrum of 519–621 nm.

For temperature shift experiments, wild-type and *scd1-1* were grown at 18°C for 7 days. Seedlings were transferred to microscope slides and then placed on the temperature-controlled stage at 25°C. Samples were imaged as described above at various times after transfer. For each reporter construct more than 5 roots were analyzed at each treatment, with representative images shown.

### PIN2-GFP and FM4-64 Analysis

Seedlings grown at 25°C were incubated in 5 μM FM4-64 to visualize endosomes (Bolte et al., 2004) for either 5 or 20 min, as indicated. The roots were rinsed in dH_2_O and imaged at various times after staining. The GFP and FM4-64 signals were visualized using a multi-track setting, as described above. No FM4-64 fluorescence was detected in the GFP channel. The FM4-64 signal was imaged using 80% laser power, a pinhole of 1.0 AU, 510 gain, and an emission spectrum of 632–759 nm. No GFP signal was detected in the FM4-64 channel. Individual images for each fluorophore and an overlay are shown with magenta for FM4-64, green for PIN2-GFP, and white for any pixels with both FM4-64 and PIN2. The red signal in these images was converted to magenta in Photoshop, changing the hue saturation. To better identify spatial colocalization independent of intensity, a thresholding function was applied to individual channels in Photoshop or in the Zeiss Zen colocalization module. We then prepared a thresholded overlay in which pixels are indicated as magenta for FM4-64, green for PIN2-GFP, and white for any pixels with both FM4-64 and GFP. To examine the change in colocalization of PIN2-GFP and FM4-64 over time, the number of PIN2-GFP, FM4-64, or both, signals in each cell with a PIN2-GFP labeled endomembrane compartment was quantified, and the percentage was determined for 740 cells from 20 roots. In the temperature shift experiments, *scd1-1* seedlings grown at 18°C for 7 days were transferred to 5 mM FM4-64 for 5 min, rinsed briefly, and then imaged at intervals of 2 min on a temperature-controlled stage at 25°C.

### Colocalization of PIN2 and Endosomal Markers

Col(g) and *scd1-1* seedlings containing PIN2-GFP were crossed with fluorescently labeled endomembrane marker lines. These constructs and their localization patterns are summarized in Supplementary Table S1. The seedlings were grown for 7 days at 25°C. Seedling roots were visualized using the Zeiss 710 laser scanning confocal microscope. GFP and RFP signals were resolved in dual channel mode with excitation at 488 and 594 nm, and emission wavelengths of 493–582 and 599–754 nm, respectively. For all samples, we verified the absence of RFP signal in the GFP channel and vice versa, using single-labeled samples. For colocalization analysis, samples were examined using Zeiss Zen software. The threshold for RFP or GFP was determined within each image, using regions that had only signal from one fluorophore. We selected regions of interest (ROI) of consistent size, focusing on individual endomembrane compartments (French et al., 2008) from at least 4 roots each of Ara6 and Ara7 reporters, measuring 21 and 17 endomembranes, respectively. Colocalization was quantified using both Pearson’s coefficients (weighted colocalization coefficients) and Manders’ overlap coefficients (Manders et al., 1993; Dunn et al., 2011).

Lines containing both PIN2-GFP and other RFP reporters were examined in dual channel mode as described above. PIN2-GFP SYP61-CFP transgenics were imaged using spectral imaging and linear unmixing at an excitation wavelength of 458 nm and a 464–648 nm filter.

## Data Availability Statement

All datasets generated for this study are included in the article/Supplementary Material.

## Author Contributions

CG and JI designed and completed the experiments and wrote the manuscript. TF, CM, DL, and KM performed the experiments and edited the manuscript. GM designed the experiments, wrote, and edited the manuscript. All authors contributed to the article and approved the submitted version.

## Conflict of Interest

The authors declare that the research was conducted in the absence of any commercial or financial relationships that could be construed as a potential conflict of interest.

## Supplementary Material

FIGURE S1 Root elongation rate is reduced after transition to the restrictive temperature. Seedlings of Col(g) and *scd1-1* were grown at the permissive temperature of 18°C and then transferred to the restrictive temperature of 25°C. Root growth was measured in real time and is plotted as a function of time after transfer and is the average 12 seedlings with average and standard error reported.

FIGURE S2 *scd1-1* has no apparent reduction in statolith abundance at either the permissive or restrictive temperature. Col(g) and *scd1-1* seedlings were grown for 5 days at 18 or 25°C, or were grown at 18°C and transferred from 18 to 25°C for 24 h before imaging. Representative images are shown from analysis of at least 5 seedlings. Scale bar = 20 mm.

FIGURE S3 In *scd1-1* grown at 25°C, PIN2-GFP and FM4-64 show limited colocalization in vesicles after a short incubation with FM4-64. PIN2-GFP (green) roots were incubated in FM4-64 (magenta) for 20 min, rinsed, and imaged immediately, where pixels of both signals are shown in white. Images were enhanced using a pseudocolor fill of a thresholded image (last panel). The figure shown is representative of the 17 roots analyzed.

FIGURE S4 PIN2-GFP and FM4-64 do not colocalize in wild-type roots. Treatment of PIN2-GFP transgenic wild-type seedlings grown at 25°C with FM4-64 for 30 min resulted in endosomes stained with FM4-64, but not PIN2-GFP.

FIGURE S5 PIN2-GFP does not colocalize with other endomembrane markers. The localization of a prevacuolar marker (SNX1-RFP, *n* > 50), a trans-Golgi marker (VHA1-RFP, *n* > 20), and a trans-Golgi/endosome marker (SYP61-CFP, *n* > 50) had limited overlap with PIN2-GFP fluorescent endomembrane bodies.

TABLE S1 Endomembrane marker lines used for localization of PIN2-GFP compartments.

TABLE S2 Summary of analysis of colocalization of PIN2-GFP and endosomal reporters within distinct endomembrane compartments.
