# Supplementary material for: A Conditional Mutation in SCD1 Reveals Linkage Between PIN Protein Trafficking, Auxin Transport, Gravitropism, and Lateral Root Initiation
Source: Front Plant Sci. 2020 Jul 7;11:910. doi: 10.3389/fpls.2020.00910 (PMC7358545; doi:10.3389/fpls.2020.00910)
Supplement: FIGURE S1 — Root elongation rate is reduced after transition to the restrictive temperature. Seedlings of Col(g) and scd1-1 were grown at the permissive temperature of 18°C and then transferred to the restrictive temperature of 25°C. Root growth was measured in real time and is plotted as a function of time after transfer and is the average 12 seedlings with average and standard error reported. [file Data_Sheet_1.PDF]

Gibson et al. Supplemental Tables and Figures

**Supplemental Table I**: Endomembrane marker lines used for localization of PIN2-GFP compartments		
SNX1-RFP	Prevacuolar compartment	(Jaillais et al. 2006)
VHA1-RFP	Trans-Golgi network	(Dettmer et al. 2006; Geldner et al. 2007)
ARA6-RFP	Late endosome or prevacuolar	(Ueda et al. 2004; Ebine et al. 2012)
RFP-ARA7	Prevacuolar compartment/ multivesicular body	(Kotzer et al. 2004; Lee et al. 2004; Haas et al. 2007; Jia et al. 2013; Cui et al. 2014)
SYP61-CFP	Trans Golgi network/ endosome	(Robert et al. 2008)

Supplemental Table II. Summary of analysis of colocalization of PIN2-GFP and endosomal reporters within distinct endomembranes

Reporter	Weighted Colocalization Coefficient	Overlap Coefficient
RFP-ARA7[1]	0.94 ± 0.02	0.82 ± 0.02
ARA6-RFP[2]	0.91 ± 0.02	0.83 ± 0.02

[1] Average and SE of 21 endomembranes after setting colocalization thresholds using Zen software. [2] Average and SE of 19 endomembranes after setting colocalization thresholds using Zen software.

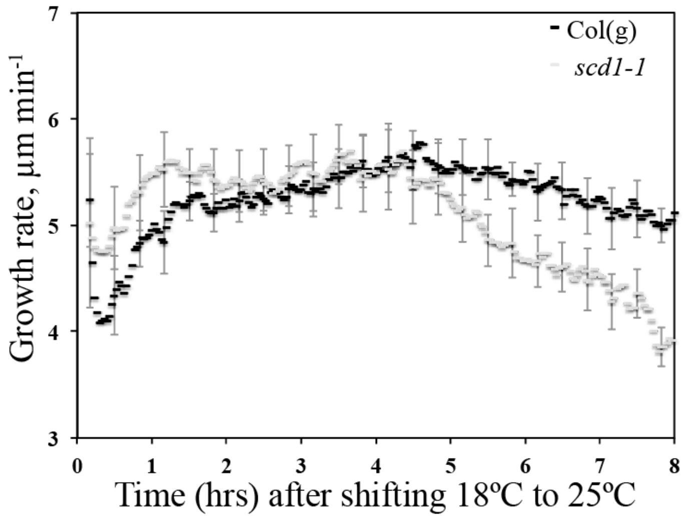

Supplemental Figure 1. Root elongation rate is reduced after transition to the restrictive temperature. Seedling of Col(g) and *scd1-1* were grown at the permissive temperature of 18°C and then transferred to the restrictive temperature of 25°C. Root growth was measured in real time and is plotted as a function of time after transfer.

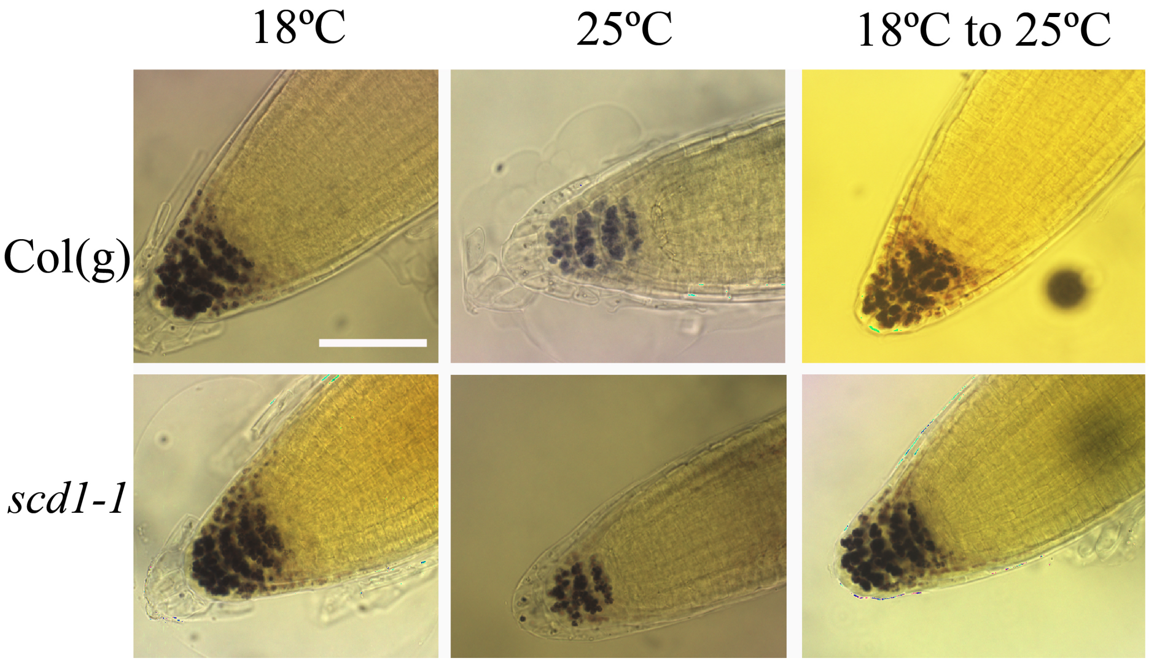

Supplemental Figure 2. ***scd1-1*** **has no apparent reduction in statolith abundance at either the permissive or restrictive temperature.** Col(g) and *scd1-1 s*eedlings were grown for 5 days at 18°C or 25°C or were grown at 18°C and transferred from 18°C to 25°C for 24 h before imaging. Scale bar = 20 µm.

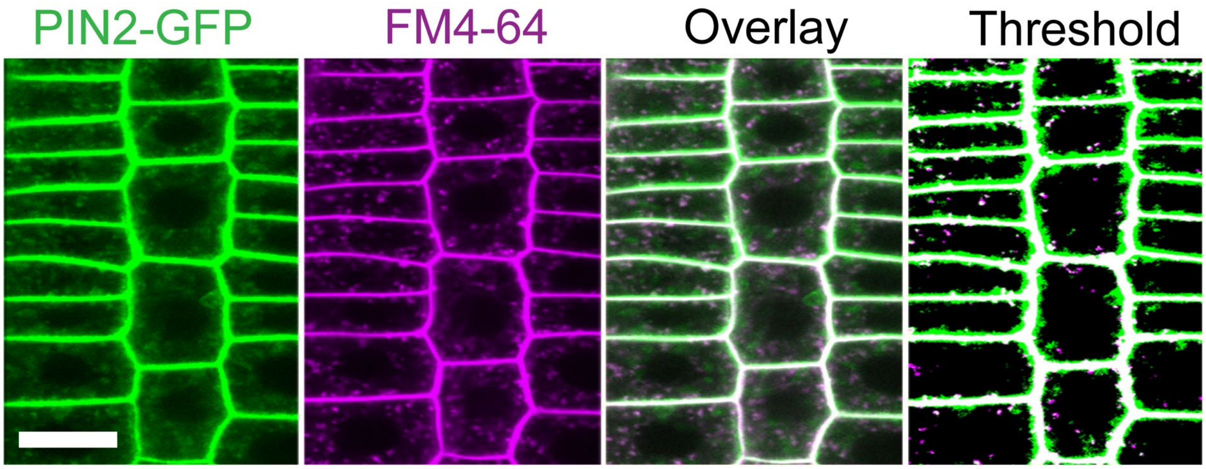

Supplemental Figure 3. In *scd1-1* PIN2:GFP and FM4-64 show limited colocalization in vesicles after a short incubation with FM4-64. A) An *scd1-1* root transformed with PIN2-GFP (green) was incubated in FM4-64 (magenta) for 20 min, rinsed, and imaged immediately, with pixels with both signals shown in white. The merged image was enhanced using a pseudocolor fill of a thresholded image (last panel). Scale bar is 10 µm.

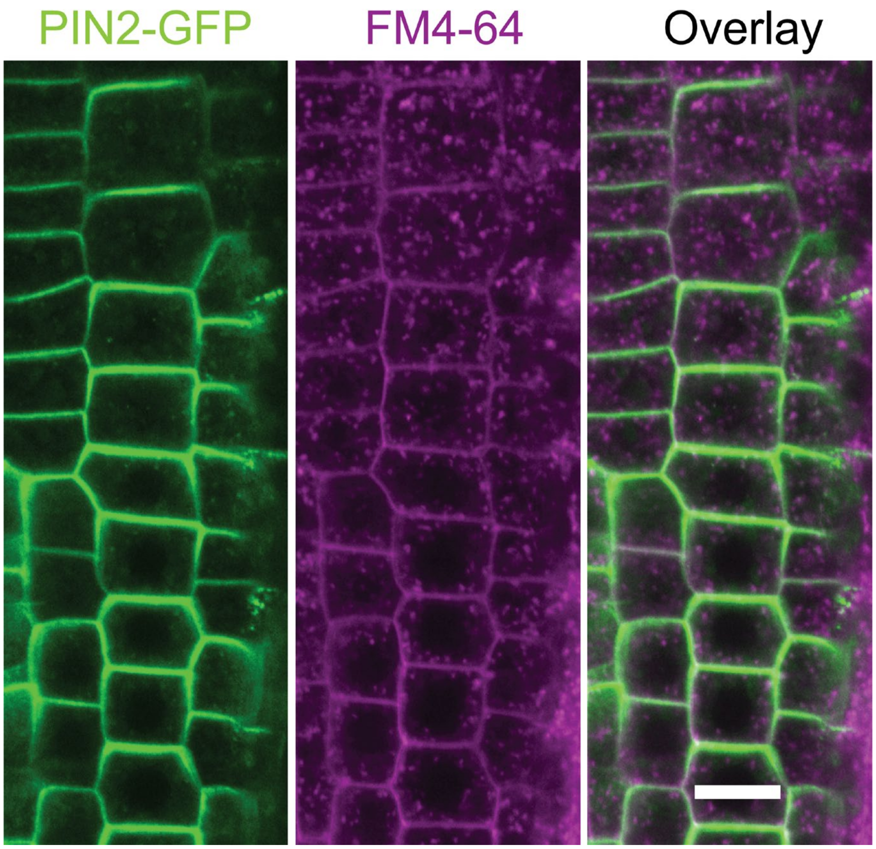

Supplemental Figure 4. In wild-type PIN2-GFP and FM4-64 show limited colocalization in vesicles after incubation with FM4-64. A) A wild-type root transformed with PIN2-GFP (green) was incubated in FM4-64 (magenta) for 30 min, rinsed, and imaged immediately, with pixels with both signals would be white. No endomembranes appear to have both both PIN2-GFP and FM4-64. Scale bar is 10 μm.

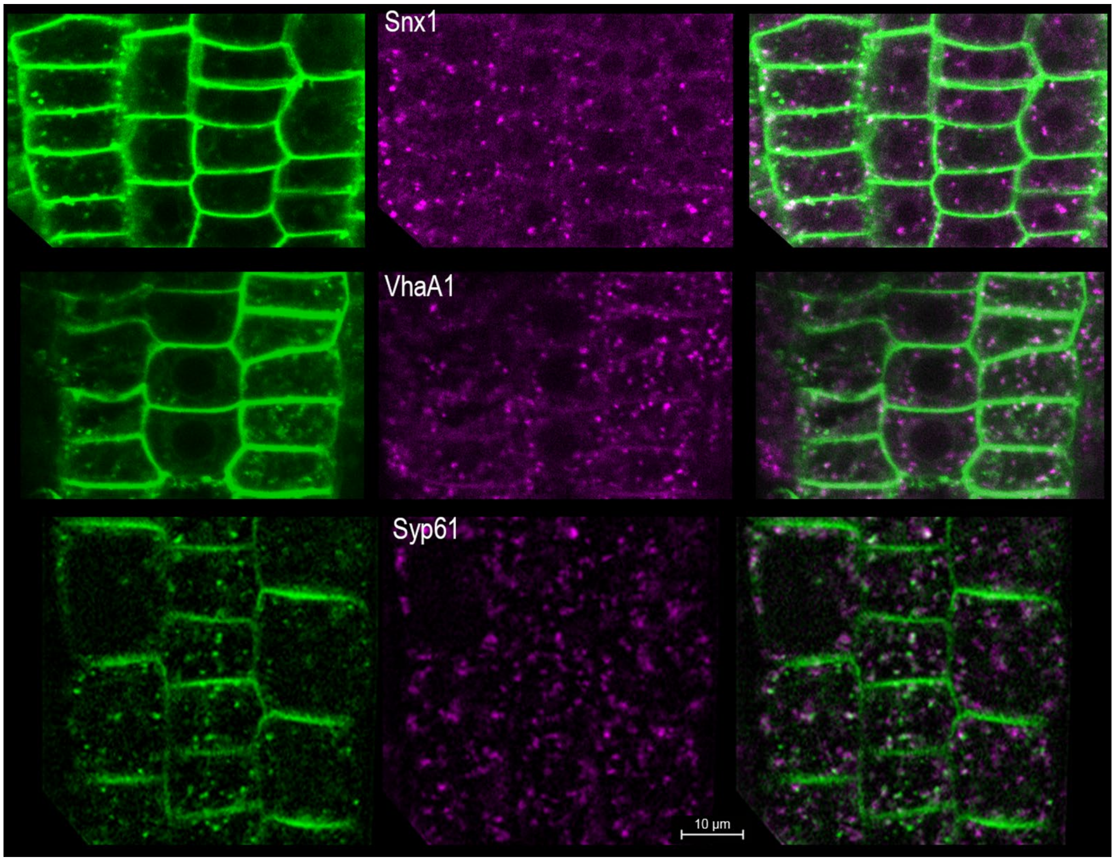

Supplementary Figure 5. PIN2-GFP does not colocalize with other endomembrane markers. The localization of a prevacuolar marker (SNX1-RFP), a trans-Golgi marker (VHA1-RFP), and a trans-Golgi/endosome marker (SYP61-CFP) have limited overlap with PIN2-GFP fluorescent endomembrane bodies.
